# The epidemic potential of avian influenza A (H7N9) virus in humans in mainland China: A two-stage risk analysis

**DOI:** 10.1371/journal.pone.0215857

**Published:** 2019-04-19

**Authors:** Xuzheng Shan, Shengjie Lai, Hongxiu Liao, Zhongjie Li, Yajia Lan, Weizhong Yang

**Affiliations:** 1 Department of Epidemiology and Biostatistics, School of Public Health, Sichuan University, Chengdu, Sichuan, China; 2 Prevention and Health Section, Affiliated Hospital, Chengdu University, Chengdu, Sichuan, China; 3 WorldPop, School of Geography and Environment, University of Southampton, Southampton, United Kingdom; 4 School of Public Health, Fudan University, Key Laboratory of Public Health Safety, Ministry of Education, Shanghai, China; 5 Flowminder Foundation, Stockholm, Sweden; 6 Chinese Center for Disease Control and Prevention, Beijing, China; 7 Department of Environmental Health and Occupational Medicine, School of Public Health, Sichuan University, Chengdu, Sichuan, China; Politecnico di Torino, ITALY

## Abstract

**Background:**

From 2013 to 2017, more than one thousand avian influenza A (H7N9) confirmed cases with hundreds of deaths were reported in mainland China. To identify priorities for epidemic prevention and control, a risk assessing framework for subnational variations is needed to define the epidemic potential of A (H7N9).

**Methods:**

We established a consolidated two-stage framework that outlined the potential epidemic of H7N9 in humans: The Stage 1, index-case potential, used a Boosted Regression Trees model to assess population at risk due to spillover from poultry; the Stage 2, epidemic potential, synthesized the variables upon a framework of the Index for Risk Management to measure epidemic potential based on the probability of hazards and exposure, the vulnerability and coping capacity.

**Results:**

Provinces in southern and eastern China, especially Jiangsu, Zhejiang, Guangzhou, have high index-case potential of human infected with A (H7N9), while northern coastal provinces and municipalities with low morbidity, i.e. Tianjin and Liaoning, have an increasing risk of A (H7N9) infection. Provinces in central China are likely to have high potential of epidemic due to the high vulnerability and the lack of coping capacity.

**Conclusions:**

This study provides a unified risk assessment of A (H7N9) to detect the two-stage heterogeneity of epidemic potential among different provinces in mainland China, allowing proactively evaluate health preparedness at subnational levels to improve surveillance, diagnostic capabilities, and health promotion.

## Introduction

The avian influenza A (H7N9) virus infections in humans since 2013 in mainland China are unprecedented both in terms of mortality and morbidity, and the extent to which the disease spread has enlarged in the wave of 2016–17 [1–3]. Previous closely monitoring of virological and molecular characteristics of A (H7N9) virus in poultry and human beings emphasizes that A (H7N9) continues to emerge and spread into populations at risk [4–9]. Therefore, the risk assessment of A (H7N9) in humans is crucial for the preparedness and response of sporadic infections, epidemic, and even potential pandemic.

The dynamics of zoonotic infections at the human-animal interface could be divided into different stages of transmission [10]. As A (H7N9) virus originates from and persists in animal reservoirs with sporadic human-human transmission [10, 11], we could divide the transmission into two stages: index-case potential, described the transition from animal hosts to human beings, resulting in an index case; and epidemic potential, characterized the subsequent widely spread of the virus in human due to exposure to poultry or human-human transmission [12]. Additionally, previous studies mainly focused on the risk assessment of index-case potential based on the *Poisson* process with the memory of past human infections or using stochastic dynamic modelling framework [13, 14]. The *Poisson* process is constrained by the piecewise linear trend [13], while the mathematical dynamic model, including susceptibility, exposure, infection, and recovery compartments is not suitable for the overdispersed data [14, 15]. Due to the lack of relevant data, multicriteria decision analysis (MCDA), a kind of knowledge-driven modelling methods, has been used as an alternative mathematical approaches to evaluate the infection risk of H5N1 [16], but a group of experts are needed to support statistical analysis for their different background and experience in the MCDA. the Boosted Regression Trees (BRT) model, a species distribution model increasingly used in ecological suitability modelling for vector-borne and zoonotic diseases [17–20], has also been used to assess the population at risk of A (H7N9). The capacity of modelling interactions between independent variables as well as non-linear relationships between the independent and predictor variables enables BRT model suitable for the risk assessment of diseases transmitted from animals to humans [21].

Moreover, the risk assessment should include the epidemiological, socioeconomic and other factors to define the epidemic potential broadly, but not only limit in animal-human level. The initiatives, i.e. the Rapid Risk Assessment of Acute Public Health Events by the World Health Organization (WHO), have reinforced a need for proactive approaches to emerging infectious diseases risk assessment [22]. The Influenza Risk Assessment Tool (IRAT), a risk assessment framework, has been used to evaluate the risk for emergence and impact dimensions [23]. However, both tools above are based on qualitative approaches, and IRAT lacks the vulnerability dimension in risk assessment. Another framework, named the FAO-OIE-WHO Four Way Linking Framework, encourages the governments to share information of human health, laboratory, epidemiology and animal health [24]. It has been commonly used at the human-animal interface but is difficult to quantify the risk. Additionally, Geerlings et al [25] developed a composite risk index to evaluate the vulnerability of avian influenza H5N1 in Egypt through questionnaires, but the assessment just includes the vulnerability of the risk together with the limitation of timeliness. Therefore, a comprehensive risk assessment framework is needed to evaluate the A (H7N9) epidemic potential proactively and quantitively, by integrating the estimates of index-case and epidemic potentials to optimize surveillance, control and treatments.

The Index for Risk Management (INFORM) model, a tool widely used for global humanitarian risk analysis by UN agencies, donors, NGOs and research institutions, defines risk through three dimensions: hazards and exposure to risks, vulnerability, and lack of coping capacity [26]. As a consolidated framework, the INFORM has been used to measure risk prospectively and quantitatively, and its output is easy to be understood for the crisis response for governments or health departments [27]. In other words, INFORM supports proactive assessment and risk management [27]. For instance, the INFORM has been applied in the pandemic potential assessment for viral haemorrhagic fever [28, 29], but it has not been used for avian influenza risk assessment. In this study, we aimed to build a unified framework based on BRT and INFORM to evaluate the risk of avian influenza A (H7N9) in humans quantitively and proactively at the provincial level. Our study may help the health departments to formulate the strategy and allocate resources for H7N9 surveillance, preventive measures, and treatments.

## Methods

### Data sources

All data were obtained from publicly available data sources, supplied and analysed in an anonymous format, without access to personal identifying information. Therefore, our study was exempt from institutional review board assessment.

The data of confirmed A (H7N9) human cases during 2013–2017 in mainland China were collated from the EMPRES Global Animal Disease Information System (EMPRES-i) of the Food and Agricultural Organization (FAO) (empres-i.fao.org/eipws3g/bioclimatic). Meteorological variables were obtained from the WorldClim database (worldclim.org/version2). The monthly temperature and precipitation were aggregated to represent annual trends, seasonality, and extreme or limiting meteorological factors. The poultry density data were obtained from Gridded Livestock of the World compiled by FAO (www.fao.org/ag/againfo/resources/en/glw/home.html). The other data included the aspects of the vulnerability and coping capacity at provincial level were collated from the yearly statistics of China between 2011 and 2016 (data.stats.gov.cn). Following INFORM approach, we normalized covariate factors and standardized to a scale of 0–10, with 10 represents the worst outcome. The data sources in the analysis are detailed in Table 1, and all codes used for these analyses are available on request from the corresponding authors.

**Table 1:** Input dataset used in the risk assessment framework.

		Data source	Website
**Stage 1**	**Index-case potential**		
	Confirmed A (H7N9) human cases	EMPRES Global Animal Disease Information System (EMPRES-i)	empres-i.fao.org/eipws3g/bioclimatic
	Environmental variables	WorldClim database	worldclim.org/version2
	Global poultry density data	Gridded Livestock of the World	www.fao.org/ag/againfo/resources/en/glw/home.html
**Stage 2**	**Epidemic potential**		
	***Exposure to risks***		
	Index-case potential Outputs of Stage 1		
	***Vulnerability***		
	Population density	National statistical data	data.stats.gov.cn
	Highway kilometers	National statistical data	data.stats.gov.cn
	Passenger capacity	National statistical data	data.stats.gov.cn
	Internet penetration	National statistical data	data.stats.gov.cn
	Teledensity	National statistical data	data.stats.gov.cn
	Poultry production	National statistical data	data.stats.gov.cn
	***Lack of coping capacity***		
	Health care institutions	National statistical data	data.stats.gov.cn
	Community health service center	National statistical data	data.stats.gov.cn

Note: A two-stage framework based on BRT and INFORM focuses on the risk of disease spread including index-case potential and epidemic potential.

### Data analysis

#### Overview

**Fig 1:**
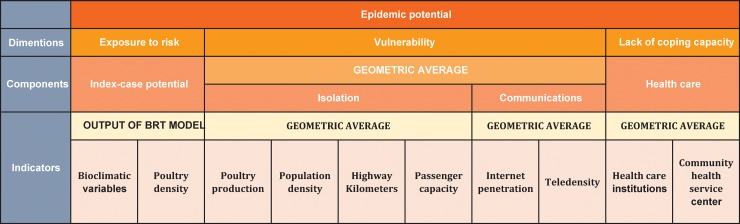
Graphical presentation of epidemic potential INFORM. Note: the exposure to risk was the index-case potential of Stage 1.

To assess the risk of avian influenza A (H7N9) infections in human beings, we developed a two-stage framework (Fig 1) based on INFORM that focused the stages of A (H7N9) virus transmission from poultry to human: Stage 1, index-case potential, described the transition from poultry to human beings, resulting in an index case; Stage 2, epidemic potential, characterized the subsequent spread of the virus in humans due to co-exposure or sporadic human-human transmission [12].

#### Stage 1: Assessing index-case potential using BRT model

The BRT model was established using the number of A (H7N9) human cases as the dependent variable. Due to the importance of poultry and climatic factors on A (H7N9) transmission [21, 30–33], the poultry density and meteorological variables were included as independent variables in the BRT model. As described in previous studies [17, 18], both presence and absence data were included to build the BRT model, and pseudo-absences were generated throughout the points in locations [34]. Pseudo-absences were twice as many as A (H7N9) human cases in the model. The data was divided into two parts, training data for building the model with 75% of cases, and testing data for evaluating the model’s goodness of fit using 25% of cases. We chose the ‘Bernoulli’ as the error structure and learning rate was 0.05, and bag fraction was 0.75, with the tree complexity of 5 as a start. Then we predicted the probability of A (H7N9) case occurrence based on the model. The area under the curve (AUC) statistic, sensitivity and Kappa were used to assess model accuracy [35]. The probability was aggregated as geomean at the provincial level and standardized on a scale of 0–10.

#### Stage 2: Quantifying epidemic potential using INFORM

We synthesized the variables upon INFORM to measure the epidemic risk from three dimensions: hazards and exposure probability, vulnerability and the lack of coping capacity [27]. In this stage, we used the results of Stage 1 as the exposure probability. Constrained by the data availability, we only collected data at the provincial level for stage 2. Therefore, we have to aggregate the outcomes of stage 1 to match the level of coarse data used in stage 2. The vulnerability is the susceptibility of communities to those hazards; lack of capacity is the shortage of resources that can help absorb the shock [26, 27]. The indicators of vulnerability included population density, poultry production, distance of highway, passenger capacity, internet penetration and teledensity [18, 32, 36]. Poultry production was different from the poultry density in stage 1. Poultry density is the density of the live chicken or ducks in natural environment, while poultry production includes eggs or chicken for people’s consumption. In stage 1, the index-case may present because of the contact with the live chicken in the farming; and in stage 2, more people may be infected through selling or buying the production in the live poultry market, therefore we used poultry density in the stage 1 and poultry production in the stage 2. The lack of coping capacity dimension included the indicators of healthcare institutions and community health service centers [27].

As the INFORM model needs at least 5 years of data to reduce variation, we used the geomeans of 2011–16 data in the model. A geometric mean is often used when comparing different items, when each item has multiple properties that have different numeric ranges. Because the factors included the probability, counts, and other types of data, the geometric means might be more suitable to describe the central tendency, and this indicator was also recommended by the INFORM [27]. The lower values of indicators, the higher risk, except poultry production, population density, distance of highway, and passenger capacity. Therefore, we standardized inputs to a 0–10 scale using the INFORM methods, with 10 represents the worst situation [27]. To aggregate the indicators, we used geometric mean and calculated the INFORM of Stage 2 by Eq 1 [27]. Fig 1 showed the graphical presentation of epidemic potential INFORM.

$$Risk={Exposure}^{\frac{1}{3}}\times {Vulnerability}^{\frac{1}{3}}\times {Lack\;of\;coping\;capacity}^{\frac{1}{3}}$$ (1)

All statistical computations were performed using R software version 3.4.3 (R Foundation for Statistical Computing, Vienna, Austria) with *dismo* and *gbm* packages.

## Results

### Overall incidence

**Fig 2:**
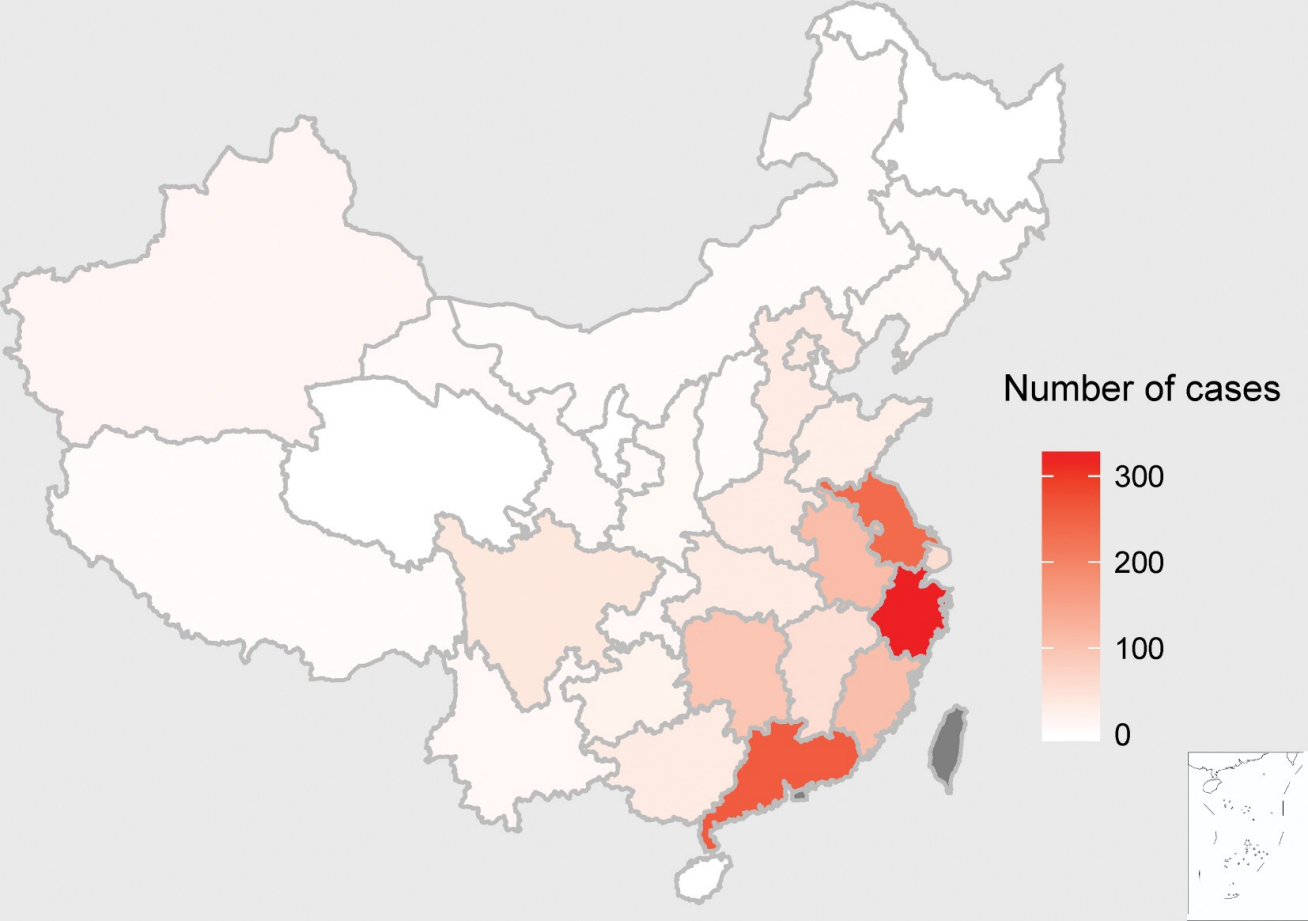
Geographic distribution of A (H7N9) cases from 2012 to 2017. Note: Data were unavailable in the grey regions in the map.

Between 2013 and 2017, a total of 1556 laboratory-confirmed human infection with A (H7N9) virus were reported in mainland China, with 145 cases reported in 2013, 314 cases in 2014, 208 cases in 2015, 130 cases in 2016 and 759 cases in 2017. The epidemic spread to most provinces except Heilongjiang, Hainan, Qinghai, and Ningxia. The cases mainly located in the Yangtze River Delta in eastern China and southern China, i.e. Zhejiang (20.81%), Guangdong (16.63%), and Jiangsu (15.35%) (Fig 2).

#### Results of Stage 1

We built a BRT model to predict the probability of A (H7N9) in humans and took 50 draws from the datasets for the uncertainty estimates. The fixed number of 1000 (IQR: 200) trees for the fitting. Mean total deviance was 1.282 (IQR: 0.008), and mean residual deviance was 0.188 (IQR: 0.017). The AUC of the training data was 0.97 (95%CI: 0.96~0.98), with Kappa was 0.54 (IQR: 0.11), and sensitivity was 0.42 (IQR: 0.10). The indicators with higher relative influence proportion were poultry density (24.59%) and mean temperature of warmest season (22.57%), precipitation of driest month (8.91%). The relative influence of the variables in the BRT model and the predicted probability of human A (H7N9) cases in mainland China are presented in Table 2.

**Table 2:** Relative influence of the variables in the BRT model (%).

variables	Relative influence (IQR)
poultry density	24.59(3.73)
Mean Temperature of Warmest Quarter	22.57(5.94)
Precipitation of Driest Month	8.91(3.26)
Precipitation of Driest Quarter	4.98(5.75)
Precipitation of Coldest Quarter	4.01(2.86)
Mean Diurnal Range	3.95(1.87)
Mean Temperature of Wettest Quarter	3.72(0.54)
Precipitation of Wettest Quarter	3.60(0.76)
Precipitation Seasonality (Coefficient of Variation)	3.50(0.44)
Precipitation of Wettest Month	2.76(0.64)
Annual Precipitation	2.33(0.68)
Precipitation of Warmest Quarter	1.90(0.37)
Mean Temperature of Driest Quarter	1.87(0.40)
Temperature Seasonality (standard deviation *100)	1.70(0.41)
Isothermality (BIO2/BIO7) (* 100)	1.58(0.22)
Min Temperature of Coldest Month	1.49(0.40)
Annual Mean Temperature	1.39(0.95)
Max Temperature of Warmest Month	1.17(0.27)
Temperature Annual Range (BIO5-BIO6)	1.00(0.15)
Mean Temperature of Coldest Quarter	0.64(0.22)

Note: The numbers showed the proportion of the influence to the probability based on the BRT model, IQR: Inter-quartile Range.

**Fig 3:**
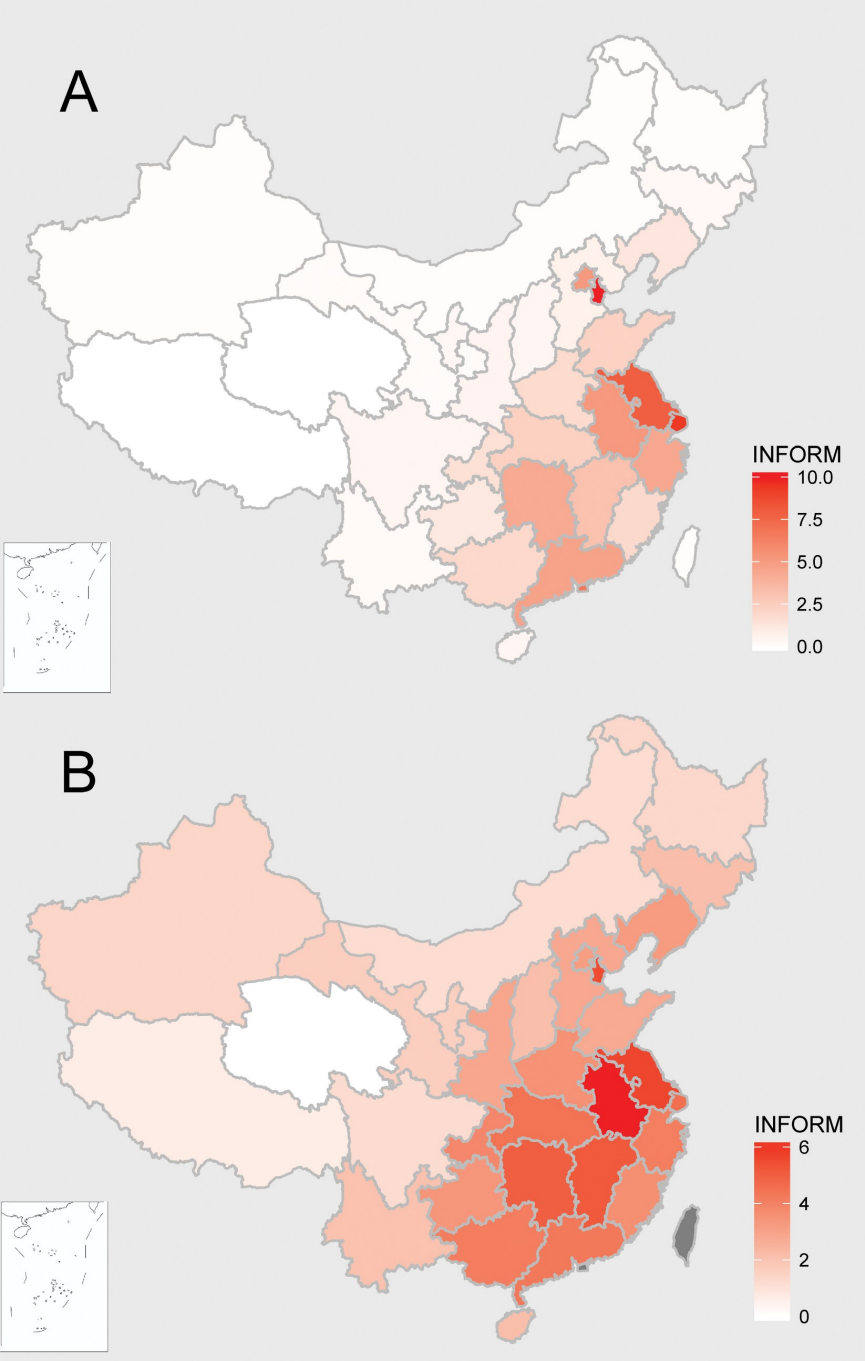
Index-case potential and epidemic potential based on INFORM. Note: (A) Index-case potential standardized by INFORM methods. (B) Epidemic potential based on INFORM. Those coloured in red had high risk and in white had low risk. Data are unavailable in the grey regions in the map.

The predicted probability at provincial level was summarized and transformed into INFORM outputs (Fig 3A). Across mainland China, the ranking of subnational regions in Stage 1, identification of locations with index cases potential, informed that the higher index potential was in coastal provinces, especially Shanghai and Jiangsu. Many locations ranking highly were in provinces with higher morbidity in 2013–17, and several provinces with lower previous morbidity had increasing index-case potential, i.e. Tianjin and Liaoning.

#### Results of Stage 2

Six provinces in all 31 provinces of mainland China had a INFORM score higher than 5, with three provinces located in central China. Overall, the score of Stage 1 was lower than that of Stage 2. The lack of coping capacity contributed more than the vulnerability for epidemic potential by INFORM. The INFORM of epidemic potential is presented in Table 3.

**Table 3:** INFORM of epidemic potential (Stage 2).

Province	Index-case potential	Vulnerability	Lack of coping capacity	Epidemic potential (Stage 2)
Overall (median and IQR)	1.2 (0.2, 3.1)	4.1 (3.1, 5.3)	7.8 (6.6, 8.8)	2.9 (1.6, 4.2)
Anhui	5.1	6.5	8.0	6.4
Jiangsu	7.9	4.7	5.0	5.7
Tianjin	10.0	1.8	9.8	5.6
Jiangxi	3.1	6.5	7.1	5.2
Hunan	4.3	6.8	4.4	5.1
Shanghai	9.4	1.1	9.5	4.6
Hubei	2.4	5.7	6.7	4.5
Guangdong	4.6	4.1	4.2	4.3
Guangxi	2.0	4.9	7.4	4.2
Zhejiang	4.4	3.0	5.3	4.1
Chongqing	1.6	4.3	8.4	3.9
Fujian	1.9	3.1	8.0	3.6
Henan	1.8	8.2	3.0	3.6
Guizhou	1.2	4.9	6.7	3.4
Liaoning	1.3	3.8	7.1	3.3
Beijing	5.1	0.7	8.4	3.2
Shaanxi	0.5	6.3	7.2	2.9
Hebei	0.7	5.0	6.6	2.8
Shandong	2.3	5.3	1.8	2.8
Hainan	0.4	2.5	9.9	2.1
Shanxi	0.4	3.7	6.9	2.1
Jilin	0.2	4.5	8.8	2.1
Yunnan	0.2	5.4	8.4	2.0
Gansu	0.1	4.1	7.8	1.6
Ningxia	0.3	1.2	9.9	1.6
Xinjiang	0.1	3.7	8.8	1.4
Heilongjiang	0.0	5.2	8.6	1.3
Sichuan	0.5	7.1	0.4	1.1
Inner Mongolia	0.1	3.2	8.4	1.1
Xizang	0.0	3.4	9.8	0.6
Qinghai	0.0	1.7	9.8	0.0

Nearly all the provinces in mainland China except Qinghai had the risk of epidemic. Central China, especially Anhui Province, stood out for the higher ranking. Central China with the most susceptible populations and poorest response capacity may be at risk of ongoing epidemic. Conversely, rankings decreased between stages in southeast China. Fig 3B showed the epidemic potential based on INFORM.

## Discussion

Based on a two-stage risk assessment framework by integrating BRT model and INFORM models, we demonstrated the locations with index-case potential and epidemic potential of human infected with avian influenza A (H7N9) virus in China. Some coastal provinces or municipalities, such as Tianjin, Liaoning, with lower former morbidity had the spillover risk, while provinces in central China had higher epidemic potential once the index case happened. According to the risk highlighted in this study, strengthening the surveillance, diagnosis capacity, and health promotion in high risk regions of China will be crucial to prevent and control the A (H7N9) epidemic.

Li et al [37] explored the risk distribution of avian influenza A (H7N9) in humans with BRT model and found that density of poultry, coverage of shrub and temperature played important roles for human H7N9 infection. Base on ecological niche models, Xu et al [32] investigated the relationships between the occurrence of H7N9 and environmental factors including meteorological variables, human population density, bird migratory routes, wetland distribution, farms, and live poultry markets, and found that the distribution of poultry, farms, and human population density were the top three determinants. Based on the evidence from previous studies, therefore, we just analysed the poultry density and meteorological factors in the index-case potential analysis and included the population density in the epidemic potential analysis. The mean total deviance of the BRT model was 1.287, and the mean residual deviance was 0.189. It means the factors included in the model could explain 85.3% of the results.

For the index-case potential defined by BRT model in Stage 1, provinces in eastern China were ranked in the higher quantile, and there was spillover risk in other coastal provinces where only sporadic cases have been reported. Most cases occurred in southeast China around Yangtze River delta, but northern coastal China even had the high incidence risk for the relative humidity and temperature. The results of BRT models showed that the precipitation, poultry density and temperature had high impacts on the index-case potential, which is consistent with Fang et al research [38]. The poultry density of northern coastal China was high, but the timing of peaks of avian influenza A(H7N9) infections in humans in northern coastal China was later than the season of southern coastal regions, which might be related to the weather factors [1]. The results suggested that A (H7N9) might spread wider than the cases having been reported, which was in line with the trend of A (H7N9) epidemic[39]. The poultry density, live poultry markets and climatic factors might all drive the high risk of A (H7N9) infections in humans. A (H7N9) virus might be undetected in poultry farms as its low pathogenicity in avian, and proactive surveillance in chicken or duck is needed to detect the A (H7N9) as early as possible [30, 40]. Live-poultry markets and poultry product trade should under suitable management, such as temporary or permanent closure of live poultry markets [41]. Additionally, eastern China with the highest numbers of A (H7N9) in humans since early 2013 should be applied with the improved diagnostic capacity to detect cases timely and accurately [1, 4].

For Stage 2, quantifying epidemic potential by using a data-informed framework, our results show that central China ranked higher than others. The high population density and mobility, frequent poultry production trade might break the isolation with southeast provinces and contribute to the vulnerability for these provinces. Our findings also suggested that socio-economic indicators might also be the driving indicators of disease spread. The recent study pointed to the fact that insufficient education, food security and income as the vulnerability of H5N1 risk [25]. Poultry markets management also play an important role in controlling the epidemic [38]. Health promotion through Internet or other effective ways is needed to make the public know about avian influenza and take recommended actions to protect themselves effectively [42, 43]. Contrast to the vulnerability, the lack of capacity had more influence to the epidemic. Strengthening the ability of diagnosis and treatment of medical institutions is also conducive to early detection and early emerging infectious disease treatment [44].

We also reported high heterogeneities across mainland China between index-case and epidemic potential of A (H7N9). Several locations, mainly in central China, ranked lowly for spillover, but were more likely to spread due to their absence of effective interventions once index-cases are present. The great increase in rank between stages emphasized the key points in which intervention strategy would play an important role.

As presented in our studies, BRT model is efficient for predicting occurrence of diseases, and has been proved to be successfully mapping the distribution of A (H7N9) virus in humans [38]. We also used the INFORM to synthesize the three dimensions quantitively. The INFORM can highlight the core of health prevention and control by ranking the index and inform the government the epidemic preparedness for key dimensions before the epidemic happens. Indeed, even when the epidemic occurs, the INFORM also can combine the modelling strategies of the two stages to address response efforts.

There are some limitations in this study. First, we used proxy covariate factors rather than specific drivers because of the data accessibility, therefore the data gaps still existed. For example, the indicator of live-poultry markets is crucial to control the epidemic [45], but we just used poultry production indicator. Second, it is hard to collect the resources at the county level, so we ranked the INFORM at the province level, but it may vary at city or county level. The third, the indicators were chosed according to the literature and limited by data availability. At last, as the clinical severity of infections and the capacity of human-to-human transmission have not changed substantially across epidemics since 2013[1], we used 5-year data to explore the index-case potential at the average level of the intensity across five waves. However, the difference of risks between the five waves might be existing, and they will be investigated in our future research. However, these limitations will be a line of investigation for the future. Although we were short of more direct and continuous data, our study also detected the at-risk provinces accordance to epidemiological stages, and applied the health departments special treatments to deal with A (H7N9).

## Conclusions

This analysis provides an integrated risk assessment framework for A (H7N9) epidemic potential in humans. The norther coastal province has the index-case potential for the relative humidity and temperature. The provinces in central China has the epidemic potential for the vulnerability and lack of capacity. The two-stage risk and its heterogeneity among provinces in mainland China detected in our study will be helpful for governments or health departments for proactively health preparedness. With the two-stage evaluation, the governments at-risk provinces should improve surveillance, diagnostic capabilities, and health promotion for the A (H7N9) transmission. The framework could also contribute to the proactive and quantitative risk assessment of other devastating pathogens.

## Supporting information

S1 Fig The predicted probability of human A (H7N9) cases in mainland China. Note: BRT model with 1100 trees was used to predict the probability of A H7N9.
(TIF)
