# Supplementary figures and images for: The epidemic potential of avian influenza A (H7N9) virus in humans in mainland China: A two-stage risk analysis
Source: PLoS One. 2019 Apr 19;14(4):e0215857. doi: 10.1371/journal.pone.0215857 (PMC6474630; doi:10.1371/journal.pone.0215857)

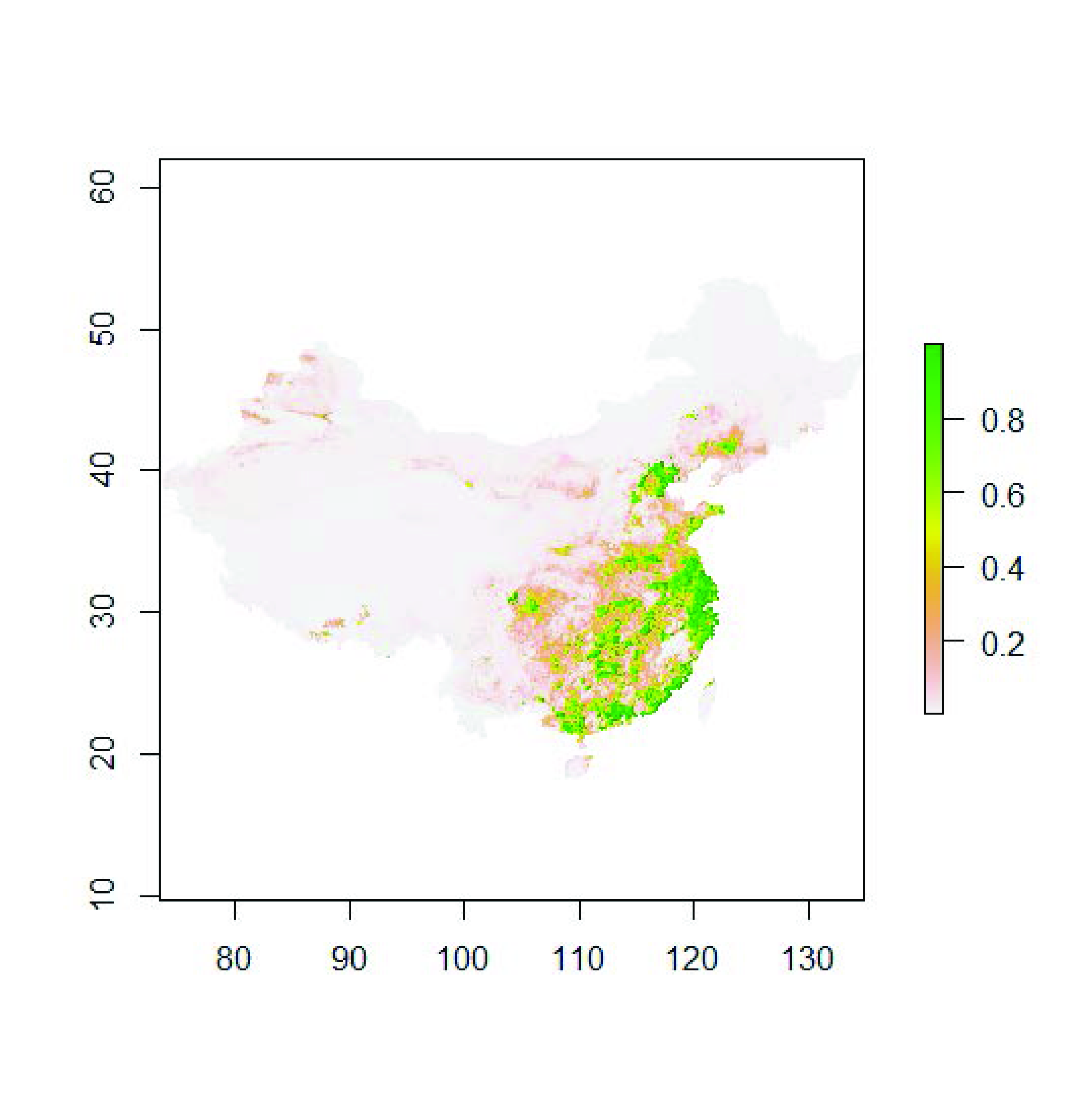

Supplement: S1 Fig — Note: BRT model with 1100 trees was used to predict the probability of A H7N9. (TIF) [file pone.0215857.s001.tif]
